# Paediatric case mix in a rural clinical school is relevant to future practice

**DOI:** 10.1186/s12909-017-1082-1

**Published:** 2017-11-29

**Authors:** Helen M. Wright, Moira A. L. Maley, Denese E. Playford, Pam Nicol, Sharon F. Evans

**Affiliations:** 1 Rural Clinical School of Western Australia, University of Western Australia, PO Box 1654, Kalgoorlie, WA 6433 Australia; 2 Division of Paediatrics, Faculty of Health and Medical Sciences, University of Western Australia, 35 Stirling Highway, Crawley, WA 6009 Australia; 3 Department of General Paediatrics, Child and Adolescent Health Service, GPO Box D184, Subiaco, WA 6840 Australia

## Abstract

**Background:**

Exposure to a representative case mix is essential for clinical learning, with logbooks established as a way of demonstrating patient contacts. Few studies have reported the paediatric case mix available to geographically distributed students within the same medical school. Given international interest in expanding medical teaching locations to rural contexts, equitable case exposure in rural relative to urban settings is topical. The Rural Clinical School of Western Australia locates students up to 3500 km from the urban university for an academic year. There is particular need to examine paediatric case mix as a study reported Australian graduates felt unprepared for paediatric rotations.

We asked:

Does a rural clinical school provide a paediatric case mix relevant to future practice? How does the paediatric case mix as logged by rural students compare with that by urban students?

**Methods:**

The 3745 logs of 76 urban and 76 rural consenting medical students were categorised by presenting symptoms and compared to the Australian Institute of Health and Welfare (AIHW) database Major Diagnostic Categories (MDCs).

**Results:**

Rural and urban students logged core paediatric cases, in similar order, despite the striking difference in geographic locations. The pattern of overall presenting problems closely corresponded to Australian paediatric hospital admissions. Rural students logged 91% of cases in secondary healthcare settings; urban students logged 90% of cases in tertiary settings. The top four presenting problems were ENT/respiratory, gastrointestinal/urogenital, neurodevelopmental and musculoskeletal; these made up 60% of all cases. Rural and urban students logged similar proportions of infants, children and adolescents, with a variety of case morbidity.

**Conclusions:**

Rural clinical school students logged a mix of core paediatric cases relevant to illnesses of Australian children admitted to public hospitals, with similar order and pattern by age group to urban students, despite major differences in clinical settings. Logged cases met the curriculum learning outcomes of graduates. Minor variations were readily addressed via recommendations about logging. This paper provides evidence of the legitimacy of student logs as useful tools in affirming appropriate paediatric case mix. It validates the rural clinical school context as appropriate for medical students to prepare for future clinical paediatric practice.

## Background

Exposure to patients is essential to clinical learning. As learners experience more clinical cases they develop illness scripts which progress their clinical reasoning and diagnostic skills [1]. Learners need to build a database of cases to assist with their clinical reasoning [2], including an appropriate case mix with a variety of diseases. A systematic review of case mix and clinical competence showed higher case exposure was positively correlated with medical students’ self-reported confidence and level of comfort [3]. One study showed improved Observed Structured Clinical Examination (OSCE) performance in students with more self-directed clinical exposure [4].

Log books have long been utilised to verify self-directed exposure to cases and to accurately reflect learning environments in undergraduate and postgraduate settings [5–8]. However the focus so far has been on students learning in urban settings.

Given the numerous reported benefits [9–12], there is international interest in expanding undergraduate and postgraduate medical teaching locations to rural contexts. It is therefore useful to demonstrate equitable case exposure and resultant learning can occur in rural relative to urban locations, particularly in paediatrics.

A US study by McCurdy and colleagues reported differences in paediatric case mix for students within the same university, but placed in different geographical locations [13]. Students at the tertiary University Medical Practice (UMP) logged more uncommon disorders and cases with allergy, diabetes and fever. In comparison, students placed in Community Private Practices (CPP) up to 475 miles (764kms) from UMP logged more routine illnesses, eye, growth and mental health problems. The most common diagnoses (excluding health supervision) were 44.6% ENT/respiratory, 8.1% well children, 6.4% skin, 5.9% gastrointestinal/urogenital, 2.1% mental health, 1% neurodevelopmental, 0.8% fever and 0.7% musculoskeletal. However, McCurdy focused on private office practice, which may be systematically different from rural practice at large. Rural practice in Australia, for example, is based on a primary care model, where cases are mostly referred to specialists by the general medical practitioner (GMP) [14].

One limited early study from Western Australia (WA) showed three-week rural selective placements of six students in paediatrics gave adequate exposure to paediatric cases compared with urban controls [15]. However, the study was of insufficient size and duration to verify quantity and quality of an appropriate case mix.

**Fig. 1:**
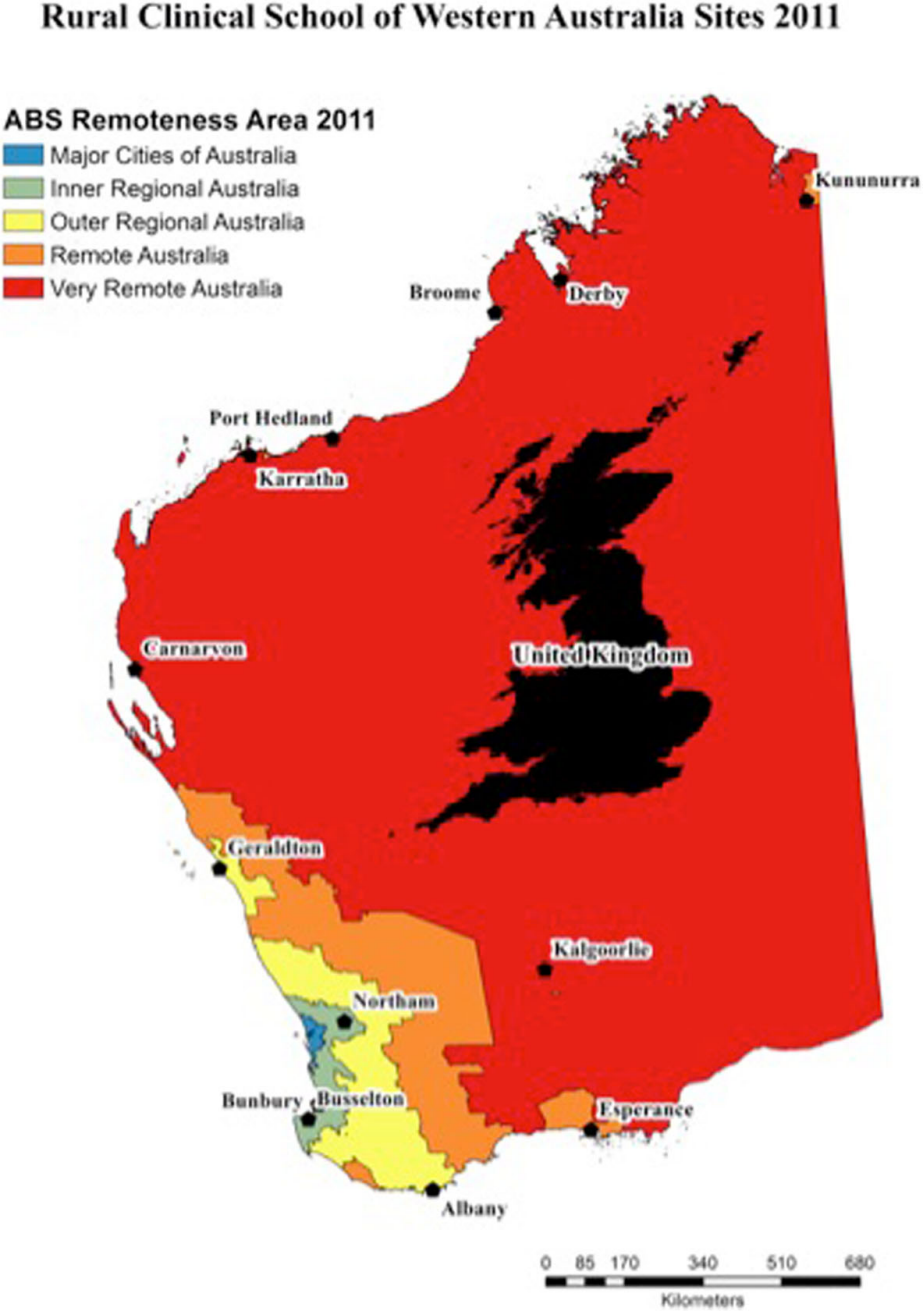
RCSWA sites in Western Australia with United Kingdom superimposed for comparison. All sites are located in outer regional, remote or very remote Australia

In WA, 25% of students undertake paediatrics in the Rural Clinical School of Western Australia (RCSWA), the remaining 75% in the urban programme, based in the state capital city. The RCSWA is well placed to provide a comprehensive overview of case logging in rural and remote contexts. This widely dispersed school locates students in 13 RCSWA sites, in groups of three to ten students, up to 2175 miles (3500 km) from the university’s city campus for an entire academic year. With respect to standardised geographical categories, all RCSWA sites are either rural (RA2-3) or remote [4, 5], shown in Fig. 1 [16]. During the forty-week rural Integrated Clerkship, students have their entire exposure to the paediatrics curriculum, making paediatrics an ideal discipline of study. The full curriculum comprised Paediatrics, Obstetrics and Gynaecology, General Practice (GP) and General Medicine concurrently. Students were predominantly community based, with access to small regional hospitals. Teaching was mostly by General Practitioners (GPs); few sites had specialist paediatric services.

Urban students undertook four separate 10-week block rotations in the same teaching disciplines as rural students. Paediatrics teaching was predominantly by specialist medical, nursing and allied health staff, based mainly in the tertiary paediatric hospital. Some placements were within secondary hospitals. All attended community placements in the capital city.

An academic paediatrician (HW) worked with the rural and urban Schools to ensure equitable learning outcomes including “primary care level of clinical skills and knowledge of the child” [17]. Rural and urban students had the same learning outcomes and completed the same summative written and OSCE assessments.

A study of Australian medical graduates suggested they felt unprepared for paediatrics in their first year of postgraduate practice [18]. Multiple factors could contribute to this, including inadequate case mix. Logs are a resource to demonstrate a paediatric case mix relevant to future clinical practice. Also to establish if paediatric case mix is comparable, despite marked geographical differences.

We therefore asked:

Does a rural clinical school provide a paediatric case mix relevant to future practice?

How does the paediatric case mix as logged by rural students compare with that by urban students?

## Methods

Logs submitted in 2011 were reviewed following written consent from medical students in their penultimate year at the University of Western Australia (UWA). Ethics approval was obtained – UWA RA/4/1/5497. Urban students’ handwritten logs were transcribed by a research assistant; rural students entered cases into a custom-built personal web database [19]. Clinical data was categorised by the lead researcher (HW), previously a rural paediatrician, currently an urban specialist general paediatrician and medical educator.

Each case was categorised into an overall presenting problem, which was compared to paediatric separations by major diagnostic category (MDC) from the Australian Institute of Health and Welfare (AIHW). AIHW is an independent statutory agency that provides annual reports based on national statistics. It uses an internationally recognised patient classification system to report on clinically meaningful patient case mix [20]. Table 1 shows categories of overall presenting problems with correlating AIHW MDC codes (excluding neonates). This indicates prevalence of paediatric presentations to public hospitals in Australia.

**Table 1:** Categories of Overall Presenting Problem with AIHW MDC codes and examples

Overall Presenting Problem allocated to student log	Examples – includes symptoms and diagnoses	AIHW MDC code
Respiratory/Ear Nose and Throat (ENT)	Cough, neck lump, stridor, otitis media	03 and 04
Gastrointestinal/urogenital	Diarrhoea, urinary tract infection, abdominal pain	06, 07, 11 and 12
Neurodevelopmental	Developmental delay, seizure, cerebral palsy	01
Musculoskeletal	Fractures, joint pain, limp	08
Skin	Rash, laceration, burn, cellulitis	09 and 22
Well child	Newborn and 6 week checks, immunisations, crying infant	24
Mental health	School refusal, anxiety, depression, autism	19 and 20
Fever	Febrile child, Kawasaki disease	Nil
Eye	Red eye, eye trauma, periorbital cellulitis	02
Growth	Obesity, short stature, failure to thrive	10
Cardiac	Congenital heart disease, murmurs, faints	05
Neonatal	Preterm, neonatal jaundice	15
Syndromes	Down’s syndrome, Williams syndrome	Nil
Allergy	Allergic rhinitis, anaphylaxis, food allergy	03
Haematology/oncology	Leukaemia, neuroblastoma, anaemia	16 and 17
Diabetes	Type 1 and Type 2 diabetes	10
Not otherwise specified	Refugee, metabolic, dental, hypothyroid	13, 14 and 21

### Inclusions and exclusions

Urban students were required to log 20 written cases, and included those from terms 2 to 4 due to logistic considerations. Rural students were required to log a minimum of 25 cases, with the option to log more. As urban students were not required to log cases in General Practice, rural cases logged in GP surgeries were excluded from the rural data set before comparison. Core Paediatric Cases from the Unit Guidebook directed logging and are listed in Appendix 1 [17].

### Protocol of data collation for analysis

The categorisation of data was based on Li et al. [8]. Age group - neonate (to 28 days), infant (to 1 year), child (2-12 years), adolescent (13-17 years); presenting complaint (e.g. cough); and organ system (e.g. respiratory) were documented.

### Case classification

The first five logged presenting symptoms of each case were entered into the data set, including the most appropriate discipline to manage that symptom. There are multiple definitions of what constitutes a generalist [21]. ‘Generalist’ was allocated if HW considered a general medical professional without specialist skills could manage the symptom. For example, an infant presenting with fever, respiratory distress and cough would have generalist entered for fever and cough but paediatric medicine entered for respiratory distress if that resulted in paediatric review or admission. A child presenting with vomiting and abdominal pain had generalist assigned for the vomiting and a paediatric surgeon for the abdominal pain if they were admitted with suspected appendicitis. Developmental assessments undertaken by students on a well child were allocated generalist when normal and developmental paediatrician when there was developmental delay. Each case was assigned a proportion of disciplines, such as 40% generalist, 20% paediatric medicine and 40% developmental.

Cases were seen in various clinical settings, coded as Levels 1 to 3. Level 1 were primary care. Level 2 were secondary care (e.g. general hospital inpatients). Level 3 were tertiary care such as sub-specialist clinics (e.g. rural visiting paediatric cardiology).

### Statistical analysis

Excel data were imported into SAS (SAS Institute Inc. C, NC, USA version 9.4). Rural-urban comparisons were made using Chi square and Fisher’s exact test (two-sided) for frequency data, and Student t-test for continuous data. Cell X^2^ was estimated to test which cells were significantly different from their expected values as a guide to interpretation. Analyses were confined to the level of the logged cases only and differences within student sets of logs were not considered relevant to the research questions.

## Results

**Fig. 2:**
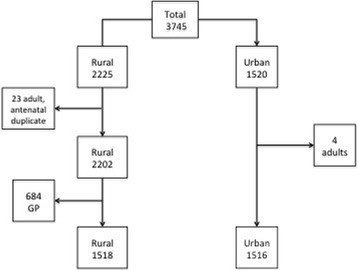
Overall cases logged for urban and rural students with exclusions

All 77 rural students consented; one withdrew and was not included. There were 145 urban students in 2011, 107 in terms 2 to 4 were eligible for recruitment and 76 (71%) consented to participate. Fig. 2 shows the 3745 cases logged with exclusions, resulting in 1516 urban and 1518 rural logs.

Rural students logged more patients seen in Level 1 and 2 healthcare settings than urban (*p* < 0.0001). The majority of paediatric cases logged by rural students were in Level 2 (91%). Urban students logged 90% of paediatric cases in Level 3, with 9% in Level 2, shown in Table 2.

**Table 2:** Healthcare settings of rural and urban logged paediatric cases

Age group	Care setting									
	Level 1 (Primary)		Level 2 (Secondary)		Level 3 (Tertiary)		Total		%	
	Rural	Urban	Rural	Urban	Rural	Urban	Rural	Urban	Rural	Urban
Neonate	8	0	181	11	6	58	195	69	12.9%	4.5%
Infant	11	2	241	21	8	303	260	326	17.1%	21.5%
Child	16	4	799	97	67	855	882	956	58.1%	63.1%
Adolescent	2	0	167	15	12	150	181	165	11.9%	10.9%
Total	37	6	1388	144	93	1366	1518	1516		
%	2.4%	0.4%	91.4%	9.5%	6.1%	90.1%				

Rural students logged more patients seen outside medical facilities such as home and school (*p* < 0.001) and more outpatients (21% v 10% p < 0.001). A higher proportion of cases were logged by urban students in emergency departments (46% v 30%, p < 0.001), as inpatients (45% v 40% *p* = 0.002) and at child development centres (2.5% v 0.9% *p* = 0.001).

### Do medical students in a rural clinical school log a case mix of paediatric patients relevant to future practice?

Rural and urban students logged core paediatric cases. The top four presenting problems of ENT/respiratory, gastrointestinal/urogenital, neurodevelopmental and musculoskeletal made up 60% of all cases logged and appeared in similar order for rural and urban, shown in Table 3. The pattern of overall presenting problems corresponded to Australian paediatric public hospital admissions. Gastrointestinal/urogenital presentations logged by all students were 16.2% compared to 16.9% AIHW, musculoskeletal 7.0% compared to 10.8%, skin 5.3% compared to 5.2% and mental health 3.8% compared to 3.5%.

**Table 3:** Overall presenting problems by age group, for rural and urban students compared to AIHW MCD codes * as percent

Rural	Neonate		Infant		Child		Adolescent		Rural total		Urban	Neonate		Infant		Child		Adolescent		Urban total		AIHW %
									n	%										n	%	
ENT / Respiratory	25	12.8%	101	38.9%	233	26.4%	25	13.8%	384	25.3%	ENT / Respiratory	16	23.2%	147	45.1%	240	25.1%	17	10.3%	420	27.7%	34.8%
Gastrointestinal/ Genitourinary	23	11.8%	36	13.9%	143	16.2%	44	24.3%	246	16.2%	Gastrointestinal/ Genitourinary	8	11.6%	48	14.7%	160	16.7%	29	17.6%	245	16.2%	16.9%
Neuro/ developmental	4	2.1%	24	9.2%	110	12.5%	14	7.7%	152	10.0%	Neuro/ developmental	4	5.8%	22	6.8%	119	12.5%	27	16.4%	172	11.3%	5.1%
Musculoskeletal	6	3.1%	3	1.2%	77	8.7%	22	12.2%	108	7.1%	Musculoskeletal	1	1.5%	4	1.2%	75	7.9%	24	14.6%	104	6.9%	10.8%
Fever	0	0.0%	22	8.5%	47	5.3%	2	1.1%	71	4.7%	Fever	6	8.7%	27	8.2%	96	10.0%	4	2.4%	133	8.8%	2.6%
Skin	6	3.1%	16	6.2%	75	8.5%	11	6.1%	108	7.1%	Skin	0	0.0%	16	4.9%	65	6.8%	11	6.7%	92	6.1%	5.2%
Well child	79	40.5%	13	5.0%	0	0.0%	0	0.0%	92	6.1%	Well child	5	7.3%	17	5.2%	25	2.6%	0	0.0%	47	3.1%	2.9%
Mental health	0	0.0%	1	0.4%	59	6.7%	19	10.5%	79	5.2%	Mental health	0	0.0%	2	0.6%	15	1.6%	20	12.1%	37	2.4%	4.0%
Other	52	26.7%	44	16.9%	138	15.6%	44	24.3%	278	18.3%	Other	29	42.0%	43	13.2%	161	16.7%	33	20.0%	266	17.5%	
Total %	195 12.9		260 17.1		882 58.1		181 11.9		1518		Total %	69 4.5		326 21.5		956 63.1		165 10.9		1516		

*excluding newborns

There were some reasonable differences between student logs and AIHW MDCs. For example, developmental assessments were included as neurodevelopmental presentations in this study, contributing to a higher proportion of student logs (11.0% compared to 5.1%). Students logged more well children and children with fever as the presenting problem compared to AIHW (4.6% v 2.9% and 6.7% v 2.6%). ENT/respiratory presentations logged by all students were lower than AIHW (27.1% compared to 34.8%); this is most likely related to student choice of cases to log.

### How does the case mix of paediatric cases logged by rural students compare to urban?

Although all students logged similar proportions of infants, children and adolescents, rural students logged significantly more neonates, (*p* < 0.001), 40% of whom were well. Urban students logged more neonates with ENT/respiratory problems than rural (23% v 12%) and more cases with a neonatal presentation (such as preterm) compared to rural (29% v 16%). No rural students logged neonates with fever. Rural students logged fewer fever cases in all age groups (*p* = 0.0001).

Rural students logged a higher proportion of patients with mental health issues (5.2%) than urban (2.4%), particularly in children aged 1 to 12 years (p = 0.0001). Rural students logged more outpatient cases with mental health issues than urban students (77% compared with 59%).

More adolescents logged by rural students had gastrointestinal/urogenital symptoms compared to urban (24% v 17%). Rural students logged more eye problems and cases with issues relating to growth; urban students logged more cases with allergy, diabetes and fever (*p* < 0.001). Fewer than one case of growth problems, allergy and diabetes was logged per student by both urban and rural students.

The number of symptoms documented by the students in their logs was analysed as an indication of morbidity (Table 4). Both groups logged the same proportions of cases with five or more symptoms, which made up nearly half the cases logged. Rural students recorded significantly more cases with only one symptom (p < 0.001).

**Table 4:** Symptoms recorded per case: cases with five or more symptoms made up nearly half the cases logged

Number of symptoms	Rural	%	Urban	%	Total	%
1	256	16.8%	76	5.0%	332	10.9%
2	197	13.0%	218	14.4%	415	13.7%
3	211	13.9%	306	20.2%	517	17.0%
4	212	14.0%	266	17.6%	478	15.8%
> = 5	642	42.3%	649	42.8%	1291	42.5%
Total	1518	100%	1515*	100%	3033	100%

* one log was removed as incomplete with no symptoms recorded

**Fig. 3:**
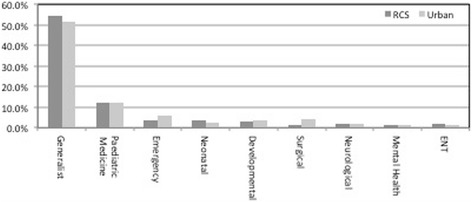
Overall proportion of discipline assigned per symptom

Each symptom was assigned as generalist or to a paediatric specialty and is shown in Fig. 3. There was no significant difference in the average proportion of generalist symptoms within the cases, with 56.7% for rural and 53.5% for urban (*p* = 0.68). The pattern of discipline distribution was similar between rural and urban, with 30% of logs having at least one symptom allocated to paediatric medicine, 11.1% to emergency medicine and 7.5% to developmental paediatrics. The only significant difference was 1.2% of rural cases and 4.2% of urban cases being predominantly general surgical (*p* < 0.0001). Cases where all symptoms were assigned as generalist comprised 29.0% of rural logs compared to 22.8% of urban logs (*p* = 0.0001).

#### Year outcomes

There was no significant difference in end of year written and OSCE assessments between urban and rural student for the 221 students who completed the academic year (*p* = 0.42 and 0.93 respectively).

## Discussion

This paper asked if students in a rural clinical school log a case mix of paediatric patients relevant to future clinical practice, and how case mix compares to urban student logs. We have shown medical students in rural and urban Western Australia logged common paediatric problems that were similar to Australian public hospital paediatric separations, despite major differences in clinical settings and without specific instruction on which cases to log. Similarities between urban and rural case mix of logs were noteworthy, with similar order and pattern by age group, and for cases with five or more symptoms. This is consistent with views Longitudinal Integrated Clerkships offer similar if not better learning opportunities, and result in similar academic performances, for paediatrics [10]. The majority of symptoms logged were those that could be managed by a generalist, aligning with the curriculum learning outcomes of medical graduates.

The top four overall presenting problems of ENT/respiratory, gastrointestinal/urogenital, neurodevelopmental, and musculoskeletal were similar for rural and urban students. The comparison with public hospital data was deliberate as AIHW MDCs indicate the paediatric presentations junior doctors encounter in WA. MDCs are also available for private hospitals, however graduates in WA generally train in the public system, and students infrequently encounter paediatric admissions in private hospitals. This data reports separations, namely admissions to hospital, so does not reflect presentations seen as outpatients. This could explain some variation between student logs and AIHW data. For example, well patients and children with fever and neurodevelopmental cases logged in outpatients or emergency departments may not be admitted to hospital.

Differences may also reflect student preferences for case logging, for example ENT/respiratory cases, as logs were not a complete record of cases seen. AIHW data being from mid 2011 to mid 2013, and inclusion of 18 year olds by AIHW may also contribute.

Minor variations between rural and urban logs reflect the relative strengths and weaknesses of the rural or urban experience and can be addressed via recommendations for student logging, structured teaching, curriculum changes, or require no change as the logs reflect clinical practice. To improve case mix, rural students were encouraged by the academic mentor to limit logs of well children. Although low in number, given the clinical importance of the presentation of the febrile child, rural students have been encouraged to log more cases with fever.

Cases with allergy, growth and diabetes problems were under-represented by rural and urban students but are covered in the structured teaching of the curriculum. Students have been recommended to log these cases to improve confidence managing them as graduates.

The majority of logged symptoms could be managed by a general medical professional without specialist skills. General surgery was the only discipline with a significant difference between rural and urban. Surgery has since been added to the rural curriculum, which should address this.

Children with mental health problems comprise a large proportion of presentations to general practice and paediatricians [22], however these were only 4% of all cases logged. Rural students logged a significantly higher proportion of mental health case presentations, which represents a strength of the rural experience in paediatrics. The difference may relate to the rural lower acuity outpatient setting relative to urban tertiary ED, where urban students logged a large proportion of their cases. Given the high prevalence of mental health issues in Australian children, especially outside major metropolitan centres [23], these presentations are important for students’ learning. Rural and urban students undertake structured teaching in child mental health, and urban students attend community placements, including child and adolescent mental health services.

Well baby checks are an important skill for medical students. Rural students logged many well baby checks. Urban students completed an obstetrics rotation immediately prior to paediatrics, where they were required to undertake baby checks. Urban students may have therefore selectively logged fewer neonates, but more neonates who were unwell. Rural students have been advised to log more unwell neonates.

### How this Australian study compares to the US study

The case mix of WA student logs generally aligned with the findings by McCurdy and colleagues. Our study extends and adds to the literature by providing a comparison of student logs of paediatric cases to national hospital separations as one component of preparation for practice. The most commonly logged case in the McCurdy study was routine health surveillance (18%), with the well child (8.1%), similar to WA rural students (6.1%).

In WA, students logged fewer ENT/respiratory, but more gastrointestinal/urogenital, neurodevelopmental and fever cases among the top presentations. These may be explained by differences in the healthcare systems between Australia and the USA, and the requirement to log all paediatric patients seen during the eight-week rotation in the McCurdy study. Since WA students had relatively few restrictions on their logging, they could be more selective with their cases.

### Limitations

Although the urban and rural students were using paper versus online logging systems respectively, the different mode of logging is unlikely to have influenced types of cases logged; the learning objectives were the same and each instrument had been tailored for the students’ learning setting with common design intent and locally driven implementation [24].

Another potential limitation includes the sampling timeframe for urban students. Although we excluded term 1, there is no reason to believe that term 1 would be different to terms 2 to 4. The choices students made of cases to log remain comparable, even though the settings were different. As the case mix was remarkably similar to AIHW admissions, this suggests rural and urban students sampled appropriately, despite not logging every case encountered. The large number of cases analysed in this study demonstrates the cases logged by urban and rural students cover the core curriculum and are comparable for the paediatric cases they are likely to encounter in hospitals as postgraduates.

## Conclusions

This study presents substantial evidence that a rural clinical school can provide an appropriate paediatric case mix in a Longitudinal Integrated Clerkship, outside a traditional urban university setting. Similarities between urban and rural logs were striking. Where there were minor differences, these were readily addressed. We have affirmed rural clinical schools as an appropriate placement for medical students to learn paediatrics relevant to future practice.

## Appendix 1

Core Paediatric Cases. Examples of paediatric cases students were expected to include as part of the curriculum

-The seriously ill child

-Acute or persistent cough, stridor and/or wheeze

-Difficulty in breathing, respiratory distress

-Vomiting

-Acute or chronic diarrhoea

-The spectrum of growth in children – failure to thrive through to obesity

-Acute or recurrent abdominal pain

-The infant who is irritable, crying and/or unsettled, or has poor feeding

-Fever and sepsis including febrile convulsion

-Rash

-Headaches

-Seizures

-Trauma including fractures, head injury, burns and scalds

-Dental trauma or maldevelopment

-Limp and structural deformities of the legs and hips

-Developmental delay

-Behavioural, learning and mental health problems including autism spectrum disorders, ADHD and depression/anxiety

-The child with a chronic health problem

-The abused child

-Common congenital problems

## Abbreviations

AIHW: Australian Institute of Health and Welfare
CPP: Community Private Practices
ENT: Ear, nose and throat
GMP: General medical practitioner
GP: General Practice
GPs: General Practitioners
MDC: Major diagnostic category
OSCE: Observed Structured Clinical Examination
RCSWA: Rural Clinical School of Western Australia
UMP: University Medical Practice
UWA: University of Western Australia
WA: Western Australia

## References

[1] Bowen JL (2006). Educational strategies to promote clinical diagnostic reasoning. N Engl J Med.

[2] Eva KW (2005). What every teacher needs to know about clinical reasoning. Med Educ.

[3] de Jong J, Visser M, Van Dijk N, van der Vleuten C, Wieringa-de Waard M (2013). A systematic review of the relationship between patient mix and learning in work-based clinical settings. A BEME systematic review: BEME guide no. 24. Med Teach..

[4] Jolly BC, Jones A, Dacre JE, Elzubeir M, Kopelman P, Hitman G (1996). Relationships between students’ clinical experiences in introductory clinical courses and their performances on an objective structured clinical examination (OSCE). Acad Med.

[5] Denton GD, DeMott C, Pangaro LN, Hemmer PA (2006). Narrative review: use of student-generated logbooks in undergraduate medical education. Teach Learn Med..

[6] Denton GD, Durning SJ (2009). Internal medicine core clerkships experience with core problem lists: results from a national survey of clerkship directors in internal medicine. Teach Learn Med..

[7] Ferenchick G, Mohmand A, Mireles J, Solomon D (2009). Using patient encounter logs for mandated clinical encounters in an internal medicine clerkship. Teach Learn Med..

[8] Li ST, Smith S, Gigante JA (2007). National survey of pediatric clerkship directors’ approaches to meeting the LCME ED-2 requirement for quantified patient criteria for medical students. Teach Learn Med.

[9] Zink T, Halaas GW, Finstad D, Brooks KD (2008). The rural physician associate program: the value of immersion learning for third-year medical students. J Rural Health.

[10] Walters L, Greenhill J, Richards J, Ward H, Campbell N, Ash J (2012). Outcomes of longitudinal integrated clinical placements for students, clinicians and society. Med Educ.

[11] Greenhill JA, Walker J, Playford D (2015). Outcomes of Australian rural clinical schools: a decade of success building the rural medical workforce through the education and training continuum. Rural Remote Health.

[12] Playford DE, Evans SF, Atkinson DN, Auret KA, Riley GJ (2014). Impact of the rural clinical School of Western Australia on work location of medical graduates. Med J Aust.

[13] McCurdy FA, Sell DM, Beck GL, Kerber K, Larzelere RE, Evans JH (2003). A comparison of clinical pediatric patient encounters in university medical center and community private practice settings. Ambul Pediatr.

[14] Royal Australasian College of Physicians. What is a physician? Available from: https://www.racp.edu.au/about/what-is-a-physician. Accessed 20 Apr 2016.

[15] Kamien M (1996). A comparison of medical student experiences in rural specialty and metropolitan teaching hospital practice. Aust J Rural Health.

[16] Australian Bureau of Statistics. Australian Statistical Geography Standard (ASGS): Volume 5 – Remoteness Structure 1270.0.55.005. Available from: http://www.abs.gov.au/AUSSTATS/abs@.nsf/Latestproducts/2C28C8B6013FB2D0CA257B03000D6DA8?opendocument. Accessed 30 May 2016.

[17] Nicol PWH, Panotidis N, Payne D. Paediatrics 5551/5552 unit guidebook. University of Western Australia: School of Paediatrics and child health; 2011.

[18] Dent AW, Crotty B, Cuddihy HL, Duns GC, Benjamin J, Jordon CA (2006). Learning opportunities for Australian prevocational hospital doctors: exposure, perceived quality and desired methods of learning. Med J Aust.

[19] Maley M, Wright H, Moore S, Auret KA (2015). Pedagogy rules: open mindset in adopting fit-for-purpose educational tools in teaching dispersed medical students. Journal of Medical Education and Curricular Development.

[20] AIHW. Australian Institute of Health and Welfare National Hospital Morbidity Separation statistics by AR−DRG (version 6.0x) 2011−12 to 2012−13. Available from: https://www.aihw.gov.au/reports/hospitals/ar-drg-datacubes/contents/data-cubes. Accessed 24 Jan 2017.

[21] Naccarella L (2014). Generalism workforce planning. Definitional, pragmatic and transformational issues. Aust Fam Physician.

[22] Hiscock H, Roberts G, Efron D, Sewell JR, Bryson HE, Price AM (2011). Children attending Paediatricians study: a national prospective audit of outpatient practice from the Australian Paediatric research network. Med J Aust.

[23] Lawrence DJS, Hafekost J, Boterhoven De Haan K, Sawyer M, Ainley J, Zubrick SR (2015). The mental health of children and adolescents. Report on the second Australian child and adolescent survey of mental health and wellbeing.

[24] Schuttpelz-Brauns K, Narciss E, Schneyinck C, Bohme K, Brustle P, Mau-Holzmann U (2016). Twelve tips for successfully implementing logbooks in clinical training. Med Teach.

